# The Roles of Social Comparison Orientation and Regulatory Focus in College Students’ Responses to Fitspiration Posts on Social Media: Cross-sectional Study

**DOI:** 10.2196/26204

**Published:** 2021-09-15

**Authors:** Kristen Pasko, Danielle Arigo

**Affiliations:** 1 Department of Psychology Rowan University Glassboro, NJ United States

**Keywords:** social media, college, fitspiration, subjective well-being, social comparison, regulatory focus, perception, well-being, young adult, college student, cross-sectional, motivation

## Abstract

**Background:**

Information shared via social media influences college students’ self-perceptions and behavior, particularly, “fitspiration” posts (ie, images of healthy food, people exercising, or fitness quotations). There are mixed findings regarding the mental health implications of fitspiration and its potential to motivate healthy behavior. Individual differences such as social comparison orientation and regulatory focus could aid in determining for whom fitspiration may be helpful versus harmful, though these characteristics have received limited attention in terms of students’ fitspiration perceptions.

**Objective:**

This cross-sectional study examined associations between students’ fitspiration use (ie, intentional versus unintentional exposure while using social media), response tendencies (ie, feelings about the self and motivation to be physically active), social comparison orientation, and regulatory focus.

**Methods:**

College students (N=344; 239/344, 69.5% women) completed an electronic survey in which they self-reported demographic information, the frequency of their social media use, exposure to fitspiration posts, typical feelings in response to fitspiration posts, and typical motivation for physical activity after viewing fitspiration posts. They also completed validated self-report measures of social comparison orientation and regulatory focus.

**Results:**

College students reported frequent exposure to fitspiration posts on social media and that they experienced negative feelings in response to these posts more often than positive feelings. Average motivation for physical activity was rated as feeling motivated “some of the time.” However, students who reported more negative feelings after viewing fitspiration also reported greater motivation to be physically active after exposure. Associations between the frequency of intentional fitspiration use and motivation for physical activity after viewing fitspiration posts were moderated by social comparison orientation (b=−0.01, *P*=.03) but not by regulatory focus (b=−0.002, *P*=.67).

**Conclusions:**

Negative feelings about the self may be motivating for students with weak social comparison orientation, as fitspiration may highlight a discrepancy between one’s real and ideal self that does not prompt dejection or disengagement. However, negative feelings for prevention-focused students might not be as motivating because there are no salient negative models to avoid. Further research into these associations is warranted and could inform future efforts to promote student health and well-being during college.

## Introduction

College is identified as a critical time for health promotion, as health decisions at this time often have implications for long-term wellness [1,2]. Social media has emerged as a promising tool for mental and physical health promotion among college students; social media is rated as a top source for information about mental and physical health during college [3], and students trust health information on social media platforms [4-6].

For example, engagement in physical activity is a critical aspect of college students’ wellness that can affect their overall physical and mental health [7]. Existing evidence indicates that social media can be both a barrier and a motivator to engaging in physical activity [8,9]. The popular “fitspiration” trend is prominent on platforms such as Instagram, which typically features images of fit individuals engaging in physical activity. These images are posted with the hashtag “#fitspiration” and are easily viewed by searching this hashtag. Posts with this hashtag are meant to inspire users to engage in physical activity and practice healthy living. For instance, these posts might include an individual in exercise clothing posing near weights or in a challenging fitness pose. Furthermore, they often include quotations such as “Be stronger than your best excuses” or “Make yourself a priority.”

Although some findings show that fitspiration does contribute to increased physical activity [10], exposure to fitspiration also has been associated with body dissatisfaction, low self-esteem, and disordered eating behaviors [11-13]. Content analyses also suggest that fitspiration posts emphasize extrinsic reasons for physical activity (ie, attractiveness and unrealistic body ideals), which are associated with negative body image and may be especially harmful for individuals at risk for disordered eating and other associated mental health concerns (eg, body dissatisfaction) [10,14]. In a sample of young adults who self-reported frequent interaction with fitspiration content, 17.4% of individuals endorsed very high levels of psychological distress and 17.7% were considered at high risk for an eating disorder [15]. Thus, viewing fitspiration images on social media can have positive or negative consequences for self-image and healthy behavior. At present, however, it is not yet clear whether certain college students are more likely to show negative (versus positive) responses to fitspiration posts. Understanding individual differences in typical responses to fitspiration among college students could inform targeted wellness promotion on social media.

The social comparison theory provides one explanation for mixed findings on the association between fitspiration and wellness [16]. It suggests that individuals evaluate themselves in a valued area or domain (eg, health or appearance) in relation to other people, which provides information about one’s current status and future standing in that domain. Individuals might view themselves as doing worse than, better than, or about the same as another individual in the comparison domain. If a discrepancy is realized between the self and others who are perceived as “doing better” in the valued domain, there may be motivation to reduce this gap by changing behavior [16]. A discrepancy between the actual and the ideal body (represented in images of fit individuals [17]) might be made salient by fitspiration posts, thus motivating healthy behaviors through a positive emotional response (eg, inspiration). However, a salient discrepancy can also prompt negative emotions and demotivation for actions toward desired fitness outcomes, as achievements similar to those of the comparison target (ie, the individual used for comparison) can seem out of reach. Fitspiration images (versus travel images) have been demonstrated to have an indirect effect on the state of body satisfaction through appearance-based social comparisons. Specifically, exposure to fitspiration was associated with more appearance comparison and this predicted lower body satisfaction [12].

Social comparison orientation reflects the degree to which individuals attend to and value social comparison information [18]. Strong comparison orientation has been reported as a potential vulnerability factor for subjective well-being, such that, for individuals who tend to highly value comparisons, subjective well-being often declines with social media use. In contrast, individuals who tend to value comparisons less often have no significant decrease in subjective well-being and sometimes experience increases in positive mental health outcomes (ie, increased self-esteem) [19]. Social comparison orientation also has been shown to moderate relations between social media use and subjective well-being, such that individuals who have a stronger (versus weaker) comparison orientation show future decreases in self-esteem and increases in loneliness and depressive feelings [20-22]. Consequently, social comparison orientation may moderate relations between these experiences and health behaviors. Yet, fitspiration literature has more often focused on the frequency of social comparisons (versus one’s tendency to compare or place greater emphasis on comparisons).

Regulatory focus, or an individual’s tendency to move toward idealized goals (ie, promotion focus) or move away from feared outcomes (ie, prevention focus), may play a similar role. Similar to work on social comparison, existing regulatory focus literature also suggests that other individuals act as a potential representation of one’s future goals or outcomes [23]. To some people (those with promotion focus), other individuals who are doing well might represent idealized goals and motivate activity, while to other people (those with prevention focus), other individuals who are doing poorly might represent feared outcomes and motivate activity.

Thus, regulatory focus represents another way to understand which individuals will be motivated or not motivated to reduce the gap between one’s perceived status and the perceived status of another individual. Tailoring weight maintenance intervention to individuals’ regulatory focus has previously outperformed self-directed weight loss efforts [24]. Furthermore, a promotion focus has been associated with better psychological outcomes than a prevention focus [25]. Individuals who scored high on promotion-focused subscales of regulatory focus measures also reported higher self-esteem and life satisfaction and lower loneliness (compared with their prevention-focused counterparts) [25]. To our knowledge, however, regulatory focus has not been examined in relation to college students’ social media use, particularly, their responses to fitspiration posts. Thus, it might be useful to apply both regulatory focus and social comparison orientation as potential influences on associations between fitspiration perceptions and subjective well-being outcomes among college students (eg, positive versus negative feelings in response to fitspiration posts or motivation for physical activity after viewing fitspiration posts).

Of note, the majority of fitspiration studies have focused almost exclusively on the health outcomes of women [14]. This aligns with body dissatisfaction research, which also tends to examine this experience in samples of women [26]. A content analysis of fitspiration images showed that women were featured more than men (68% and 31%, respectively) [27]. Furthermore, women in fitspiration posts were more likely to be showing skin or to be portrayed in sexualized ways, while men were more likely to be shown as muscular [27]. Mental health outcomes for men related to fitspiration are just now beginning to receive attention. Some early research has suggested a potential increase in negative mood and a decrease in body satisfaction among men due to exposure to fitspiration or thinspiration (ie, images emphasizing thinness in models), in addition to an increase in the urge to improve their own muscularity [28]. Overall, there is a need to further examine fitspiration use and response among both college women and men.

Toward these ends, the first aim of this cross-sectional study was to describe college students’ overall social media use, their reported frequency of exposure to fitspiration posts, and their reported “typical” responses to these posts (ie, positive versus negative feelings and motivation for physical activity). The second, and ultimate, aim was to understand whether social comparison orientation or regulatory focus moderates associations between fitspiration exposure and responses. We thus aimed to identify the type(s) of students who might be most vulnerable to negative effects of fitspiration exposure while controlling for gender.

We hypothesized the following:

1. Social comparison orientation will moderate associations between feelings about the self after fitspiration exposure and self-reported physical activity motivation after fitspiration, such that those with stronger (versus weaker) social comparison orientation will show a stronger positive association.
2. Regulatory focus will moderate associations between feelings about the self and self-reported physical activity motivation after fitspiration, such that those with a greater promotion focus will show a stronger positive association than those with a greater prevention focus.
3. Social comparison orientation will moderate associations between both intentional and unintentional fitspiration exposure and self-reported physical activity motivation after fitspiration, such that those with stronger (versus weaker) social comparison orientation will show a stronger positive association.
4. Regulatory focus will moderate associations between both intentional and unintentional fitspiration exposure and self-reported physical activity motivation after fitspiration, such that those with a greater promotion focus will show a stronger positive association than those with a greater prevention focus.

## Methods

### Participants and Procedures

As part of a larger study of college students’ experiences, students enrolled at the supporting institution (who were ages 18-23 years) were recruited to participate in an electronic survey about students’ social habits and social media use. Data were collected between 2017 and 2019. In exchange, they received psychology course credit at a small university in the northeastern United States. Students used an online scheduling website to access available research opportunities; those interested in the study were directed to an online survey and instructed to complete it at their convenience. Data were deidentified after participants were assigned course credit. Procedures were approved by the respective institutional review board, and informed consent was documented electronically. The sample consisted of 336 students (235/336, 69.9% women; mean age 19 years; mean body mass index [BMI] 24.0 kg/m^2^). Additional demographic information can be found in Table 1; these characteristics are reflective of the population at the supporting institution [29].

**Table 1:** Demographic characteristics of participants.

Characteristic			Frequency (N=344)^a^, n (%)
**Age**			235
	18	115 (48.9)	
	19	80 (34)	
	20	26 (11)	
	21	11 (4.7)	
	≥22	3 (1.3)	
**Gender**			344
	Men	105 (30.5)	
	Women	239 (69.5)	
**BMI^b^ (kg/m^2^)**			338
	<18.5	9 (2.7)	
	18.5-24.9	225 (66.6)	
	25-29.9	73 (21.6)	
	≥30	31 (9.2)	
**Race/ethnicity**			344
	Black/African American	11 (3.2)	
	White/Caucasian	275 (80)	
	Latino/Hispanic	23 (6.7)	
	East Asian	0 (0)	
	South Asian	16 (4.7)	
	Native American	7 (2)	
	Multiracial	12 (3.5)	
**Year in school**			344
	Freshman	232 (67.4)	
	Sophomore	76 (22.1)	
	Junior	23 (6.7)	
	Senior	7 (2)	
	>4 years	6 (1.7)	

^a^Some participants did not answer all questions. Therefore, the total for each variable category differs.

^b^BMI: body mass index.

### Measures

#### Demographic Information

Participants were asked to indicate their age, gender, racial/ethnic identification, living situation, height, and weight. Reported height and weight were used to calculate BMI (kg/m^2^).

#### Social Media Use

To describe students’ general social media use, participants were asked to self-report their frequency of using social media platforms with options of “less than one day per week,” “1-2 days per week,” “3-4 days per week,” “5-6 days per week,” “once per day,” and “more than once per day.” Platforms included Facebook, Snapchat, Instagram, Pinterest, YouTube, Twitter, blog sites, and LinkedIn.

#### Fitspiration Exposure

To better understand students’ exposure to the fitspiration trend on social media, questions were included to assess days per week intentionally and unintentionally viewing posts, with intentional and unintentional viewing as separate items. Response options included “less than one day per week,” “1-2 days per week,” “3-4 days per week,” “5-6 days per week,” “once per day,” and “more than once per day.” We asked participants for feelings about the self after viewing posts with a 5-point Likert scale from 1 (“much better than before viewing”) to 5 (“much worse than before viewing”). Motivation to engage in physical activity after viewing posts was measured with a 5-point Likert scale from 1 (“never”) to 5 (“all of the time”). These items were used in a previous study of fitspiration perceptions among posters and followers [30].

#### Social Comparison Orientation

The Iowa-Netherlands Social Comparison Measure [18] comprises 11 items that assess general social comparison. It includes questions such as “I always pay a lot of attention to how I do things compared with how others do things.” Responses are rated on a 5-point Likert scale from 1 (“I strongly disagree”) to 5 (“I strongly agree”) and are summed to create subscale scores; higher scores indicate a stronger tendency to compare socially. This measure has shown strong psychometric properties among college students, and internal consistency has previously been cited as *α*=.83 [18]; in this study, *α*=.82.

#### Regulatory Focus

The General Regulatory Focus Measure [31] uses 18 items to assess respondents’ tendencies to approach positive outcomes versus avoid negative outcomes (9 promotion items and 9 prevention items). The promotion-focused portion includes statements such as “I frequently imagine how I will achieve my hopes and aspirations,” while the prevention-focused portion includes items such as “In general, I am focused on preventing negative events in my life.” Responses are provided on a 9-point Likert scale ranging from 1 (“not at all true of me”) to 9 (“very true of me”). Items are summed, and the prevention score is subtracted from the promotion score, with higher scores indicating greater promotion focus. This measure also has shown strong psychometric properties, including internal consistency for promotion items (*α*=.81) and prevention items (*α*=.75) [31]. In this study, internal consistency was *α*=.89 for promotion items and *α*=.77 for prevention items.

### Statistical Analysis

To address the first study aim, descriptive statistics were obtained for the reported frequency of social media use across platforms, social comparison orientation, regulatory focus, and the 4 variables related to fitspiration: (1) frequency of intentional and (2) unintentional exposure to fitspiration, (3) typical feelings about the self after viewing fitspiration posts, and (4) typical level of motivation to engage in physical activity after viewing fitspiration posts. Descriptive differences between genders in the variables of interest were obtained with two-tailed independent samples *t* tests corrected for unequal group sizes. Pearson correlation coefficients were used to examine bivariate relations between these experiences. To address the second study aim, separate general linear models were used to test for the potential moderating effects of social comparison orientation and regulatory focus on associations between (1) average feelings after viewing fitspiration and average motivation for physical activity after viewing fitspiration, (2) intentional fitspiration use and average motivation to engage in physical activity after viewing fitspiration, and (3) unintentional fitspiration use and average motivation to engage in physical activity after viewing fitspiration. Sensitivity analyses were used to determine the potential impact of gender on interpretation of moderation models. Statistical significance was set at *P*<.05, and effect sizes were expressed as change in *R^2^*. Our sample size of 336 students afforded power >.80, allowing us to detect small effects with these tests.

## Results

### Social Media Use, Fitspiration Exposure and Response, and Individual Differences in Social Comparison Orientation and Regulatory Focus

Snapchat was the most frequently used platform among students, with the majority of participants (309/344, 89.8%) reporting use at least once per day. Following in popularity was Instagram (277/344, 80.5%), Facebook (190/344, 55.2%), Twitter (122/344, 35.5%), and YouTube (120/344, 34.9%). Participants reported spending a mean of 2.95 hours per day (SD 2.23) on these platforms.

On average, intentional searches for fitspiration appeared to be less common (at least 5-6 days per week), compared with unintentional exposure (at least once every day). On average, students’ typical motivation for engaging in physical activity after viewing fitspiration posts was reported as “occasional” (mean 3.09, SD 1.01; Table 2). Feelings about the self after viewing fitspiration posts were predominately “about the same” or “somewhat worse” than prior to viewing (mean 3.40, SD 0.85).

Overall, significant gender differences were seen in the majority of the variables of interest. Compared with men, women reported greater frequency of unintentionally viewing fitspiration posts (t_318_=−2.87, *P*=.01). Women indicated more negative feelings about the self (t_224_=−6.71, *P*<.001) and higher motivation, on average, after viewing fitspiration (t_158_=−2.57, *P*=.01). Lastly, women endorsed a stronger social comparison orientation, (t_335_=−2.10, *P*=.04). No significant gender differences were found in intentional fitspiration use (*P*=.39) or regulatory focus (*P*=.97). Age was not associated with any variables of interest (intentional fitspiration use, *P*=.12; unintentional fitspiration use, *P*=.15; feelings about the self after fitspiration, *P*=.06; physical activity motivation after fitspiration, *P*=.32; regulatory focus, *P*=.55; social comparison orientation, *P*=.67). Race/ethnicity was not associated with the variables of interest (intentional fitspiration use, *P*=.05; feelings about the self after fitspiration, *P*=.99; physical activity motivation after fitspiration, *P*=.14; regulatory focus, *P*=.18; social comparison orientation, *P*=.06), with the exception of unintentional fitspiration use (*P*=.02). However, no pairwise comparisons were significant in a Tukey post hoc test (*P*=.08-.99). As controlling for gender did not meaningfully affect our results or conclusions, we presented the results of reduced models, without gender, for parsimony.

**Table 2:** Correlations between fitspiration use and response variables.

Variable			Intentional fitspiration		Unintentional fitspiration		Feelings after fitspiration		Physical activity motivation after fitspiration		Social comparison orientation		Regulatory focus
**Intentional fitspiration**													
	*r*	1		0.544		–0.168		0.384		–0.013		0.073	
	*P* value	—^a^		<.001		.003		<.001		.814		.199	
**Unintentional fitspiration**													
	*r*	0.544		1		0.042		0.247		0.005		0.128	
	*P* value	<.001		—		.452		<.001		.924		.023	
**Feelings after fitspiration**													
	*r*	–0.168		0.042		1		–0.046		0.144		–0.130	
	*P* value	.003		.452		—		.401		.008		.018	
**Physical activity motivation after fitspiration**													
	*r*	0.384		0.247		–0.046		1		0.038		0.045	
	*P* value	<.001		<.001		.401		—		.486		.414	
**Social comparison orientation**													
	*r*	–0.013		0.005		0.144		0.038		1		–0.165	
	*P* value	.814		.924		.008		.486		—		.003	
**Regulatory focus**													
	*r*	0.073		0.128		–0.130		0.045		–0.165		1	
	*P* value	.199		.023		.018		.414		.003		—	

^a^Not applicable.

As shown in Table 2, social comparison orientation was positively associated with feelings about the self after viewing fitspiration (*r*=0.14*, P*=.008), such that students with a stronger (versus weaker) comparison orientation experienced more frequent negative feelings. In contrast, regulatory focus was inversely associated with feelings about the self after viewing fitspiration, such that students with a stronger promotion focus experienced more frequent positive feelings (*r*=−0.13, *P*=.02). In addition, regulatory focus was positively associated with unintentional fitspiration use, such that students with a stronger promotion focus more frequently encountered fitspiration posts without searching for them (*r*=0.13, *P*=.02). No significant associations were found between motivation to engage in physical activity after fitspiration and either social comparison orientation (*P*=.49) or regulatory focus (*P*=.41).

Intentional fitspiration use was negatively associated with feelings about the self (*r*=−0.17, *P*=.003), and positively associated with motivation to engage in physical activity after viewing fitspiration (*r*=0.38, *P*<.001). However, unintentional fitspiration use was only associated with motivation (*r*=0.25, *P*=.001). Feelings and motivation after fitspiration were not correlated with one another (*r*=−0.05, *P*=.40).

### Moderating Roles of Social Comparison Orientation and Regulatory Focus

**Figure 1:**
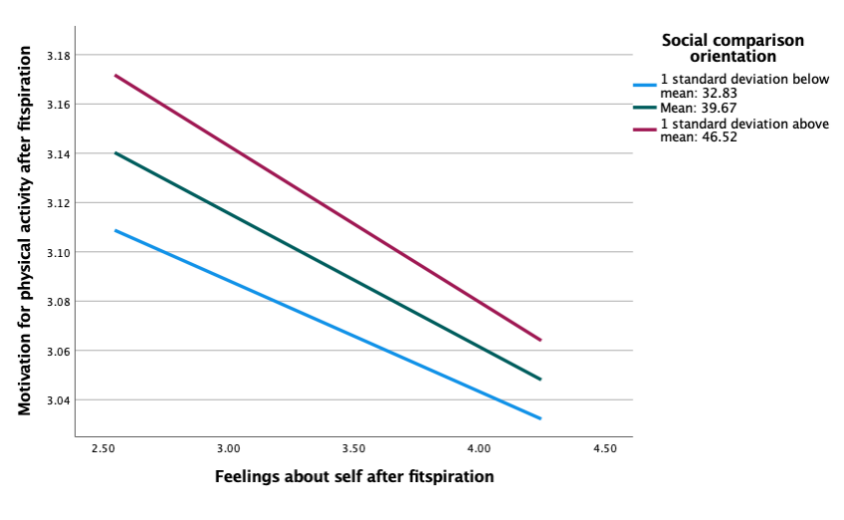
Moderation of feelings about self and motivation for exercise after fitspiration by social comparison orientation.

**Figure 2:**
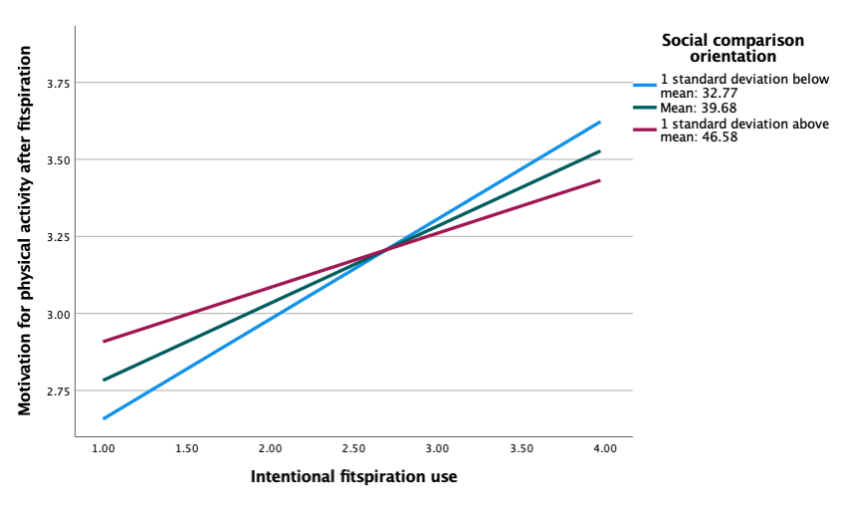
Moderation of intentional fitspiration use and motivation for exercise after fitspiration by social comparison orientation.

**Figure 3:**
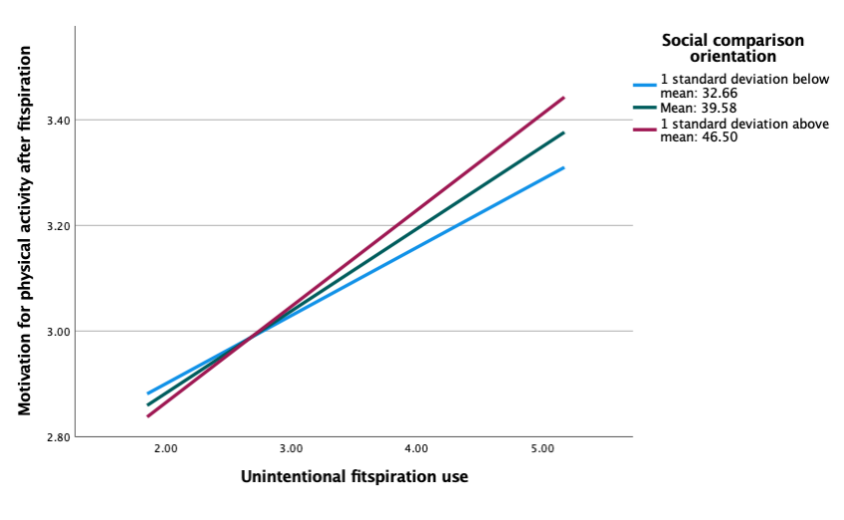
Moderation of unintentional fitspiration use and motivation for exercise after fitspiration by social comparison orientation.

As indicated, our ultimate aim was to examine potential individual differences in associations between fitspiration exposure and responses based on social comparison orientation and regulatory focus. Social comparison orientation did not moderate associations between feelings about the self and motivation for physical activity after viewing fitspiration (b=−0.001, *P*=.88; Table 3). At all levels of social comparison orientation, there were insignificant negative associations between average feelings and average motivation after viewing fitspiration (Figure 1). However, social comparison orientation did moderate the association between intentional fitspiration use and motivation after viewing fitspiration (b=−0.01, *P*=.03; Table 4). Adding this interaction resulted in a significant change in *R*^2^ (Δ*R*^2^ =.016, *P*=.03). There were positive linear associations across all levels of comparison orientation, with the strongest association in those with the highest level of comparison orientation (Figure 2). This association was not significant when fitspiration use was unintentional, however (b=0.003, *P*=.41; Figure 3).

**Table 3:** Moderation of associations between feelings about self and motivation for physical activity after fitspiration by social comparison orientation.

Variable	Motivation for physical activity after fitspiration				
	b	SE	95% CI	*t* test (df)	*P* value
Feelings after fitspiration	−0.0012	0.38	−0.74 to 0.74	−0.003 (329)	.10
Social comparison orientation	0.008	0.03	−0.05 to 0.07	0.25 (329)	.80
Feelings after fitspiration × social comparison orientation	−0.001	0.009	−0.02 to 0.02	−0.15 (329)	.88

**Table 4:** Moderation of associations between fitspiration use and motivation for physical activity after fitspiration by social comparison orientation.

Variable	Analysis 1: motivation for physical activity after intentional fitspiration						Analysis 2: motivation for physical activity after unintentional fitspiration				
	b	SE	95% CI	*t* test (df)	*P* value	b		SE	95% CI	*t* test (df)	*P* value
Fitspiration use	0.67	0.19	0.30 to 1.1	3.5 (310)	<.001	0.004		0.18	−0.36 to 0.37	0.02 (309)	.98
Social comparison orientation	0.03	0.01	0.001 to .06	2.1 (310)	.04	−0.01		0.02	−0.05 to 0.03	−0.55 (309)	.58
Fitspiration × social comparison orientation	−0.01	0.005	−0.02 to 0.001	−2.2 (310)	.03	0.004		0.005	−0.01 to 0.01	0.82 (309)	.41

**Figure 4:**
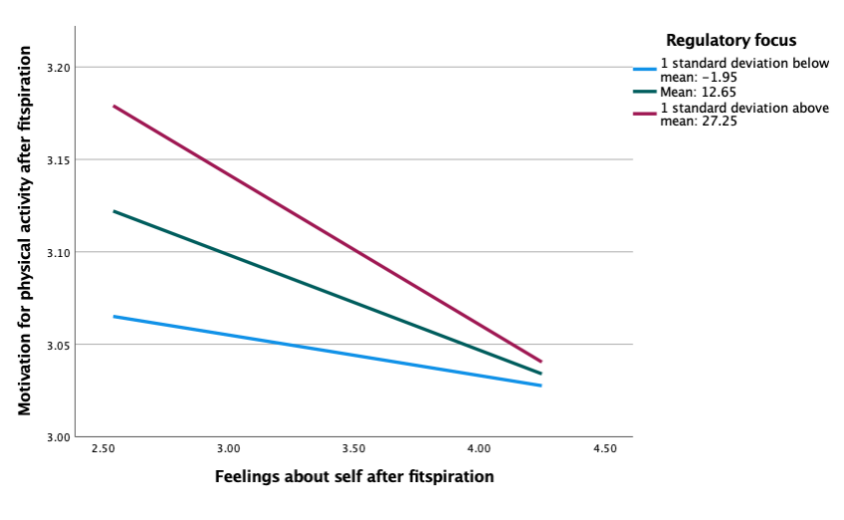
Moderation of feelings about self and motivation for exercise after fitspiration by regulatory focus.

**Figure 5:**
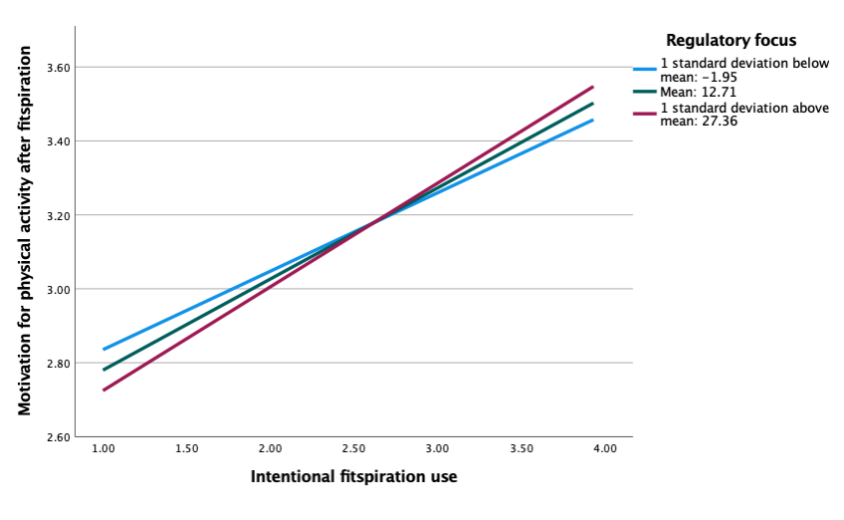
Moderation of intentional fitspiration use and motivation for exercise after fitspiration by regulatory focus.

**Figure 6:**
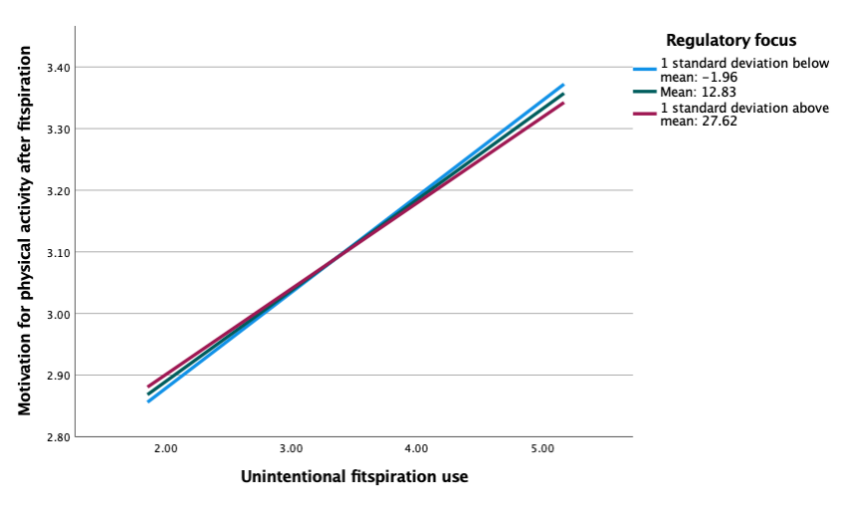
Moderation of unintentional fitspiration use and motivation for exercise after fitspiration by regulatory focus.

Regulatory focus also did not moderate the association between feelings about the self and motivation for physical activity after viewing fitspiration (b=−0.002, *P*=.67; Table 5). Simple slopes were similar, overall, for models with social comparison orientation and regulatory focus (Figure 4). However, there were larger differences between levels of regulatory focus compared with social comparison orientation. Almost no association was present at low levels of regulatory focus, compared with low levels of social comparison orientation, which showed stronger negative associations. In moderation analyses between fitspiration use (intentional and unintentional) and motivation for physical activity after fitspiration, simple slope patterns in regulatory focus were once again similar to that of social comparison orientation (ie, strong positive associations across all levels), although there were no significant differences (*P*=.33-.83; Table 6 and Figures 5-6).

**Table 5:** Moderation of associations between feelings about self and motivation for physical activity after fitspiration by regulatory focus.

Variable	Motivation for physical activity after fitspiration				
	b	SE	95% CI	*t* test (df)	*P* value
Feelings after fitspiration	−0.03	0.08	−0.19 to 0.14	−0.31(328)	.76
Regulatory focus	0.01	0.02	−0.02 to 0.04	0.53 (328)	.59
Feelings after fitspiration × regulatory focus	−0.002	0.05	−0.01 to 0.01	−0.42 (328)	.01

**Table 6:** Moderation of associations between fitspiration use and motivation for physical activity after fitspiration by regulatory focus.

Variable	Analysis 1: motivation for physical activity after intentional fitspiration						Analysis 2: motivation for physical activity after unintentional fitspiration				
	b	SE	95% CI	*t* test (df)	*P* value	b		SE	95% CI	*t* test (df)	*P* value
Fitspiration use	0.22	0.05	0.13 to 0.31	4.7 (309)	<.001	0.15		0.04	0.07 to 0.24	3.5 (311)	.001
Regulatory focus	0.006	0.01	−0.02 to 0.01	−0.94 (309)	.35	0.001		0.01	−0.01 to 0.02	0.022 (311)	.83
Fitspiration × regulatory focus	0.002	0.002	−0.002 to 0.01	0.98 (309)	.33	−0.001		0.002	−0.05 to 0.003	−0.26 (311)	.79

## Discussion

### Principal Findings

The overall purpose of this study was to examine college students’ self-reported exposure and response to fitspiration posts (with respect to their cognitive and emotional responses) and examine individual differences in psychological tendencies that could distinguish between helpful versus harmful fitspiration use.

Intentional searches for fitspiration posts appeared to be less common than unintentional exposure to these posts (ie, while scrolling through a newsfeed). This finding seems intuitive, given that individuals might more commonly be exposed to fitspiration posts randomly while scrolling through their newsfeed, rather than possessing previous knowledge of the fitspiration trend and purposefully searching for these posts. Average feelings about the self after viewing fitspiration posts were predominately “about the same” and “somewhat worse” than feelings about the self prior to viewing. Motivation for physical activity after viewing these posts was low to moderate. These findings are important due to their direct contrast with the stated intentions of the fitspiration trend, which is to inspire positive self-image and behaviors [30,32].

Overall, women self-reported significantly more unintentional viewing of fitspiration posts, more negative feelings about the self, greater motivation for physical activity after viewing posts, and greater social comparison orientation, compared with men. The observed gender difference in mood after viewing fitspiration posts could be due to factors such as (1) greater focus on women in fitspiration posts; (2) less social acceptance in identifying negative moods among men; or (3) a tendency among men to only report negative moods after viewing muscular or bare-chested images, while women appear to have negative reactions regardless of image detail [33]. However, controlling for gender did not affect any of our conclusions.

Social comparison orientation was positively associated with feelings about the self after viewing fitspiration, while regulatory focus was negatively associated with feelings about the self after viewing fitspiration and positively associated with unintentional fitspiration use. Both results are consistent with previous literature, which shows that a stronger (versus weaker) social comparison orientation is associated with worse self-reported mood after viewing others’ social media posts [34]. Previous research also suggests that individuals with a promotion focus (ie, a focus on meeting goals versus avoiding failures) tend to be motivated when seeing others who represent a higher achievement of a goal, relative to the individual’s current state (ie, positive role models) [31]. Interestingly, the present findings show that these individuals are also more likely to view fitspiration unintentionally. It is possible that highly promotion-focused individuals naturally have a cognitive bias toward focusing on and remembering these positive role models from their social media experiences. Existing work demonstrates that even when social media posts have a positive tone, individuals with a stronger social comparison orientation still report more negative mood (compared with when they view neutral posts or no posts) [35]. As the target may be different across social comparison orientation and regulatory focus, this raises a question about potential cognitive biases within both of these processes.

Of note, students who reported more negative feelings about the self after viewing fitspiration also reported more motivation to engage in physical activity. Consistent with social comparison theory, the increased salience of a discrepancy between an individual’s body, appearance, or activity level and that of another individual may promote body dissatisfaction and motivation for physical activity, to reduce a perceived gap between the self and other [36]. In this study, individuals with stronger (versus weaker) comparison orientations did show trends toward stronger associations between negative feelings and motivation for physical activity after viewing fitspiration posts, though the moderating effect of comparison orientation was not significant.

This is somewhat inconsistent with previous literature that suggests it might be more harmful to the mental well-being of individuals with high social comparison orientation to view comparison-inducing stimuli (ie, stimuli regarding self-esteem or self-evaluation) on Facebook as well as on Instagram [20]. Social comparison orientation has also been cited as mediating associations between Instagram use and a variety of mental health outcomes (eg, physical appearance anxiety, depressive symptoms, body dissatisfaction) [37]. It is possible that social comparison orientation does not differentiate individuals with whom fitspiration may be more harmful versus helpful. However, there are some important considerations. Previous studies examined social media use broadly, rather than use specific to the fitspiration trend, and the few studies that were in this area focused on differentiating between comparisons made toward “better-off” and “worse-off” others. Consequently, the discrepancy between findings could be a function of measurements used. Specifically, previous work may not have captured individuals’ stable perceptions of how important social comparison information is to them. It also might be that what truly differentiates harmfulness versus helpfulness of fitspiration is how important *certain kinds* of social comparison information are to an individual (ie, those who you perceive are doing better than you) rather than social comparison information more generally. It is also possible that inconsistencies in these findings are due to the methods used in this study. For example, since there are no validated measures of fitspiration exposure and response, we used items generated for our work in this area. It is possible that these measures were not sensitive to the differences of interest and that the use of different measures would have led to different outcomes.

In addition, students who had more negative feelings about the self after viewing fitspiration also reported more motivation to engage in physical activity after viewing fitspiration, regardless of the level of social comparison orientation. These associations held for those with greater regulatory focus (ie, strong promotion focus or equal promotion and prevention focus), but the associations did not hold for lower regulatory focus (ie, strong prevention focus). Thus, (negative) feelings about the self after viewing fitspiration may play a role in motivating physical activity across all levels of social comparison orientation. When individuals are higher in regulatory focus (ie, focus that is predominately or partly concentrated on promotion—an outlook of “I want to meet my goals”), negative feelings about the self might be motivating in a similar way. However, this is not the case with people low in regulatory focus (ie, focus that is predominately concentrated on prevention— an outlook of ”I want to prevent my failures”). Although there is minimal literature examining the role of regulatory focus in fitspiration use, previous literature has suggested that “positive” role models (similar to an upward social comparison) might be motivating for individuals with a high regulatory focus (ie, promotion-focused individuals) but not motivating for physical activity for individuals low in regulatory focus (ie, prevention-focused individuals); instead, “negative” role models might be more motivating for these individuals [31,38].

Neither social comparison orientation nor regulatory focus were associated with intentional fitspiration use. Social comparison orientation did significantly moderate associations between intentional fitspiration use and motivation after viewing fitspiration, though the effect size was weak. This was not the case for the same model with unintentional fitspiration use. Regulatory focus was not a significant moderator of either type of fitspiration use. Although not significant, patterns for the moderation analyses of social comparison orientation and regulatory focus were nearly identical for the associations between average motivation to engage in physical activity after viewing fitspiration and (1) intentional fitspiration use and (2) unintentional fitspiration use. Simple slopes across all models suggested strong positive linear associations between variables, at all levels of each moderator. It might be useful to further examine the common and distinct contributions of social comparison orientation and regulatory focus within fitspiration to clarify potential individual differences.

Finally, despite significant gender differences in the majority of variables of interest, sensitivity analyses for all moderation models suggested that the inclusion of gender as a covariate did not meaningfully change interaction effects. It is possible that this was due to a smaller sample size for men than women. However, although some differences may exist in fitspiration use, general patterns in response may persist across gender. This would be consistent with emergent research suggesting some overlap in how men and women interact with fitspiration posts [33,35]. Specifically, it has been posited that, similar to women, men experience some negative effects of fitspiration images. However, images that were most harmful for men, specifically, included heavily muscular and bare-chested males. Furthermore, men have been suggested to have less of a poor response to appearance-based social comparisons, potentially due to (1) a lower frequency of comparisons, (2) a fear of making comparisons and seeming less “manly,” (3) a decreased frequency of making upward-comparisons, or (4) a tendency to feel less negatively than women do after making comparisons [33].

Overall, this cross-sectional study provides additional support for the observations that (1) individuals who tend to have negative feelings about the self after fitspiration also report greater motivation for physical activity, (2) social comparison orientation moderates associations between intentional fitspiration use and motivation for physical activity after fitspiration, and (3) considerable overlap appears to be present across levels of social comparison orientation and regulatory focus in moderating associations between feelings about the self and motivation for physical activity after fitspiration use. However, these findings should be interpreted with caution until replicated.

### Implications of This Study

Recently, safety concerns have been raised in response to the increasingly prevalent nature of mental and physical health symptoms on social media, such as depression and disordered eating behaviors [39]. Some argue that social media provides a supportive space to share health experiences (eg, weight loss efforts) or receive a response to a plea for help [40]. Others argue that these platforms allow for perpetuation of negative health behaviors. Before #fitspiration, #thinspiration led to controversy about the glamorization of images that suggested eating disorders. The thinspiration hashtag has since been banned to prevent further harm [41], and individuals attempting to use it are redirected to mental health resources. This study’s findings demonstrate the power of fitspiration in promoting negative feelings, which may be an unintended consequence of the trend. Although negative feelings might ultimately lead to positive health behaviors (eg, physical activity motivation), more nuanced examination of fitspiration and similar trends is needed to determine for whom and under what circumstances these trends are beneficial (versus harmful).

### Strengths, Limitations, and Future Directions

Strengths of this study were its considerable sample size for detecting the hypothesized effects and its novel examination of social comparison orientation and regulatory focus as potential individual differences in fitspiration outcomes. However, as an initial investigation, this study was limited by the self-report methods used to assess fitspiration exposure and response; although these fitspiration assessments have been used in previous work, they have not been validated using traditional psychometric evaluation. Recruitment for this study occurred between 2017 and 2019; some fitspiration trends in exposure and response might have changed since collection, given the rapidly changing nature of social media and the introduction of platforms such as TikTok. Other limitations include a cross-sectional design that does not allow for determining the direction of observed effects. Using temporally sophisticated methods to clarify the sequence of platform use and behaviors (eg, longitudinal or within-person designs) would be informative, as would experimental methods to determine cause-and-effect relations between fitspiration exposure and response. Given the sample’s heavy inclusion of individuals that identify as Caucasian, female, and of healthy BMI, results should be interpreted with caution and should be replicated across more diverse samples. Different racial and ethnic groups promote different body ideals (eg, there are larger body ideals among Black women versus White women [42]). Therefore, these results should be interpreted with caution, as they may not generalize for people of color. Meta-analyses have also suggested that research in areas such as body satisfaction has been biased toward the experiences of Caucasian women and their body ideals (ie, the thin ideal) [43]. Thus, future fitspiration and body image research should take care to avoid generalizing from homogenous samples. Additional work is needed to determine whether social comparison tendencies may help to identify students most vulnerable to the negative effects of fitspiration, particularly, with a more heterogenous sample.

### Conclusions

Findings from this study show associations between college students’ fitspiration exposure and subjective well-being (ie, feelings about the self and motivation for physical activity), with potential moderators to identify individuals who might have helpful versus harmful mental health effects of fitspiration. Students who reported more negative feelings, on average, after viewing fitspiration also reported greater motivation for physical activity after exposure. Social comparison orientation did not significantly moderate associations between feelings about the self and motivation for physical activity; this requires further exploration. Social comparison orientation and regulatory focus appeared to function similarly within fitspiration use. This is with the exception of negative feelings about the self potentially acting as a motivator for individuals with a low social comparison orientation, as fitspiration may produce a discrepancy between one’s real and ideal self, while negative feelings for individuals with a low regulatory focus (ie, prevention-focused individuals) might not be as motivated because there are no negative outcomes to avoid. A greater understanding of these associations within the context of fitspiration could allow for a maximization of health benefits and a minimization of harm from social media use among college students.

## Abbreviations

BMI: body mass index.
